# Proteins: interaction at a distance

**DOI:** 10.1107/S2052252515020217

**Published:** 2015-10-30

**Authors:** Roman A. Laskowski, Janet M. Thornton

**Affiliations:** a European Bioinformatics Institute (EMBL-EBI), Wellcome Genome Campus, Hinxton, Cambridge CB10 1SD, UK

**Keywords:** protein–protein interactions, protein flexibility, bound and unbound protein forms

## Abstract

How do the surface side chains of a protein behave when it binds to another protein? Do they optimize interactions by crumpling inwards or by extending outwards?

Proteins are rather social molecules, cooperating and networking with others, interacting with DNA and RNA, nestling in cellular membranes, and working closely with small metabolites. They rarely act alone, and some are even highly promiscuous (Nobeli *et al.*, 2009 ▸). So, how do proteins greet one another when they meet (Fig. 1 ▸ *a*)? Do their surface side chains remain largely rigid as they shake hands (Fig. 1 ▸ *c*), do they crumple up against each other like the limbs of crushed rush-hour commuters (Fig. 1 ▸ *b*), or are their welcomes more distant and standoffish (Fig. 1 ▸ *d*)?

The question is addressed by Chakravarty *et al.* (2015 ▸) in this issue of **IUCrJ**. The authors examined the protein–protein interfaces of 281 bound–unbound protein structure pairs from the University of Utrecht’s Protein–Protein Interaction Affinity Database (Kastritis *et al.*, 2011 ▸). These protein pairs constitute a non-redundant set of protein pairs for which the three-dimensional structures are known not just of the complex, but also of each individual protein on its own (*i.e.* not in a complex). Also known are the dissociation constants for each complex. By comparing the unbound structures (U) of each protein with their bound equivalents (B) – minus the binding partner – the authors were able to analyse the conformational changes that occur on binding.

The principal observation was that in 69% of the cases the accessible solvent area (ASA) of the interface atoms was higher in the B form than in the U form. It is as though the surface side chains of each binding partner are reaching out towards one another in greeting as the two proteins dock (*i.e.* as in Fig. 1 ▸ *d*). The authors call this the ‘partner attraction effect’. Conversely, in the remaining 31% of the cases, the side chains withdraw to optimize their interactions; the ‘partner accommodation effect’. However, it is rare for both partners to behave in this shrinking violet manner, and at least one tends to make the more forward approach. In fact, in nearly 90% of the cases either one or both of the proteins reaches out in welcome to the other.

These stretching conformational changes allow the side chains to feel out where they can optimize contacts or make hydrogen bonds with their opposite numbers. For example, a rigid replacement of either protein with its unmodified U form reduced the numbers of interface hydrogen bonds by about 45%. Other conformational changes were also observed, although not as marked.

Fig. 1 ▸ emphasizes the small size of the side chains relative to the bulk shape of the protein. Unlike arms that can embrace partners, the side chains are like rather short fingers, which change their conformations slightly on complexation. Consequently, most of the changes observed in this dataset are actually quite small, with much variation between complexes. The exceptions to this statement are likely to be found in the ‘order-to-disorder’ transitions, which could not be captured in this study.

In conclusion, it seems that although these social molecules reach out to one another in friendly greeting as they approach, the geometry of the polypeptide backbone dictates that for many proteins they cannot properly embrace, but rather enjoy close contact without too much flexibility.

## Figures and Tables

**Figure 1:**
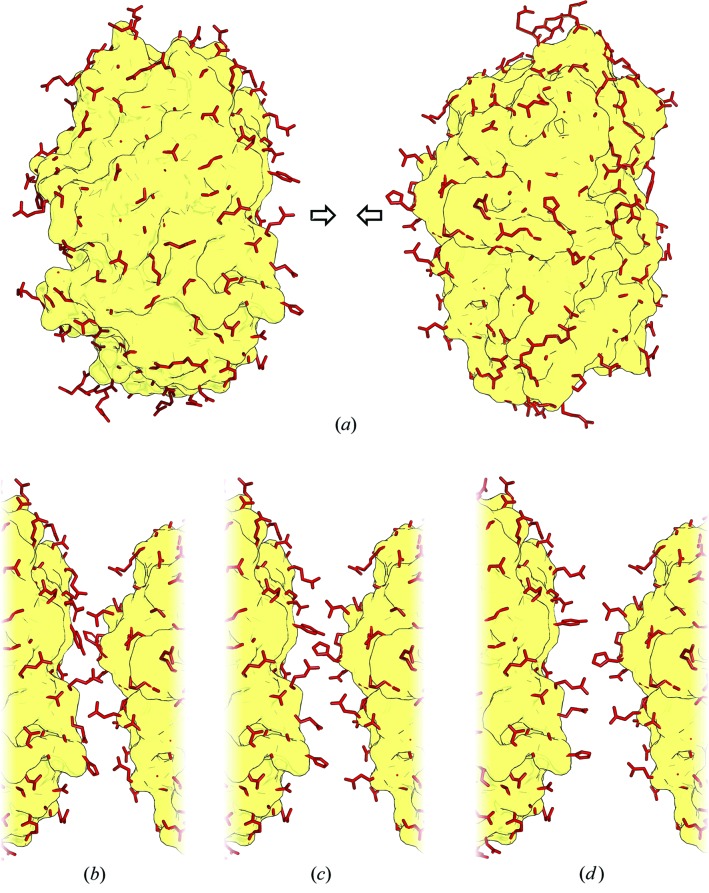
Schematic diagram showing possible behaviours of surface side chains when two proteins interact. (*a*) The two proteins are shown in surface representation with their solvent accessible surface side chains depicted as red sticks. (*b*) Compact interaction, where the side-chain conformations fold inwards as the proteins dock together. (*c*) Rigid interaction, in which the side-chain conformations hardly alter, in terms of solvent accessibility, from their conformations in (*a*). (*d*) Extended interaction, with the side chains stretching out to greet their partners as the proteins approach.
